# A Human Head and Neck Squamous Cell Carcinoma Cell Line with Acquired *cis*-Diamminedichloroplatinum-Resistance Shows Remarkable Upregulation of BRCA1 and Hypersensitivity to Taxane

**DOI:** 10.1155/2011/521852

**Published:** 2011-10-20

**Authors:** Yuriko Saiki, Takenori Ogawa, Kiyoto Shiga, Makoto Sunamura, Toshimitsu Kobayashi, Akira Horii

**Affiliations:** ^1^Department of Molecular Pathology, Tohoku University School of Medicine, Sendai, Miyagi 980-8575, Japan; ^2^Department of Otolaryngology-Head and Neck Surgery, Tohoku University School of Medicine, Sendai, Miyagi 980-8574, Japan; ^3^Department of Digestive Tract Surgery and Transplantation Surgery, Tokyo Medical University Hachioji Medical Center, Hachioji, Tokyo 193-0998, Japan

## Abstract

Recently, an inverse relationship between resistance to platinum-based chemotherapeutic agents and taxanes has been implicated in breast and ovarian cancers, and a possible pivotal role for BRCA1 has also been suggested. Because *cis*-diamminedichloroplatinum (CDDP) and taxanes are the most active antitumor agents against head and neck squamous cell carcinoma (HNSCC), we analyzed the sensitivity of nine HNSCC cell lines and their previously established derived CDDP-resistant cell lines to two representative taxanes: docetaxel and paclitaxel. None of the nine original cell lines showed any cross resistance between CDDP and taxanes, but one of the CDDP-resistant cell lines, RPMI2650CR, demonstrated hypersensitivity to both taxanes when compared to the parental cell line, RPMI2650. Furthermore, RPMI2650CR exhibited increased expression of BRCA1. These data suggest that (i) taxanes are a good candidate for a second-line therapeutic drug for HNSCC patients with acquired CDDP resistance and (ii) BRCA1 can be a candidate marker for predicting an inverse CDDP/taxane sensitivity phenotype in HNSCC.

## 1. Introduction

Cisplatin (*cis*-diamminedichloroplatinum, CDDP), a DNA damaging drug, is the most active antitumor agent for treating patients with head and neck squamous cell carcinoma (HNSCC). However, acquired resistance to CDDP is one of the major problems in the clinical management of such patients [1]. CDDP-resistance has been reported to be associated with decreased cellular accumulation of the drug, increased levels of glutathione, DNA repair, and antiapoptotic activity [2]. Hence we focused on factors or molecules that are closely associated with sensitivity and/or resistance to CDDP, and we identified *IGF2* as one of the candidate molecules for acquired CDDP resistance [3]. Ultimately, we need to find a way to deduce the molecular mechanisms for acquisition of resistance. In the meantime, it is also very important to find alternative drug(s) or method(s) for defeating the acquired resistance. Stordal et al. highlighted an inverse relationship between CDDP and taxane resistance [4]. These drugs function in different phases of the cell cycle; thus CDDP and taxanes are often applied as combined chemotherapy [5, 6]. Herein, we studied the inverse relationships of resistance between CDDP and taxanes in HNSCC cell lines and observed that one of our cell lines with acquired CDDP resistance showed increased taxane sensitivity when compared with its parental cell line. Furthermore, dramatic upregulation of BRCA1 was also observed in these CDDP-resistant cells. Although our findings are just the tip of the iceberg and many other mechanisms are yet-to-be-discovered, it is possible that the BRCA1 status can be used as one of the biomarkers to forejudge the taxane sensitivity of HNSCC with acquired CDDP resistance.

## 2. Materials and Methods

### 2.1. Cell Lines

Nine human HNSCC cell lines were used; the origins of the cell lines are one nasal cavity cancer (RPMI2650), two maxillary cancers (HSQ89, IMC4), and six oral-cavity cancers (HSC2, HSC3, HSC4, Ca9-22, HO-1-u-1, SAS). These cells were grown by methods indicated by the original developers. We also established their derived CDDP-resistant (CR) cell lines by a method described elsewhere [7].

### 2.2. MTT Assay

A total of 5 × 10^3^ cells/well was seeded in each well of flat-bottomed 96-well plates in triplicate and cultured in 200 *μ*L of medium. After 18 hours of incubation, either CDDP, or paclitaxel or docetaxel were added to the media using a fivefold dilution series, and the cells were cultured for 48 hours. Then the medium was replaced with 100 *μ*L of 0.05% 3-[4,5-dimethylthiazol-2-yl]-2,5-diphenyl-tetrazo-lium bromide (MTT)/PBS and incubated for one hour. After the incubation, the MTT solution was removed, and the cells were suspended in 100% ethanol. Absorption was measured at 590 nm using a Versamax microplate reader (Amersham Biosciences Corp., Piscataway, NJ). Independent triplicate assays were performed. All values represent the mean ± standard deviation (SD) from 3 sets of independent cultures.

### 2.3. Western Blotting Analysis for BRCA1

A total of 2 × 10^5^ cells were harvested, and protein concentrations in total cell lysates were measured using a DC protein assay kit (Bio-Rad, Hercules, CA). A 40 *μ*g aliquot of the protein was subjected to immunoblotting using a 10–20% polyacrylamide gradient gel (Bio-Rad, Hercules, CA). Antibodies used were rabbit anti-BRCA1 monoclonal antibody (Oncogene, Boston, MA), mouse anti-*β* actin monoclonal antibody (Sigma, St Louis, MO), and horseradish peroxidase conjugated anti-mouse immunoglobulin antibodies (Amersham Biosciences Corp., Piscataway, NJ). For blocking conditions and concentrations of antibodies, we followed the manufacturer's recommendations. Signals were visualized by reaction with ECL Detection Reagent (Amersham Biosciences Corp., Piscataway, NJ) and digitally processed using LAS 1000 Plus with a Science Lab 99 Image Gauge (Fuji Photo Film, Minamiashigara, Japan).

### 2.4. Statistical Analysis

The statistical differences in cell viability between the parental and CDDP-resistant cell lines were determined with student's *t*-test, and the significant difference level was established at *P* < 0.05.

## 3. Results and Discussion

In a previous study, we successfully established CDDP resistant cell lines from all of the HNSCC cell lines we attempted [3]. These results may indicate that acquisition of CDDP chemoresistance is not a rare event; we frequently observe such acquired resistance in HNSCC patients undergoing chemotherapy in the clinical setting. Hence it is important to understand the mechanisms of acquired resistance to CDDP, as well as trying to find alternative methods for clinical management of patients with acquired CDDP resistance. We also identified *IGF2* as one of the candidate genes for acquired CDDP resistance in HNSCC by cDNA microarray analysis, and the stably *IGF2*-expressing RPMI2650 cells did show resistance to CDDP similar to that observed in RPMI2650CR [3]. 

**Figure 1:**
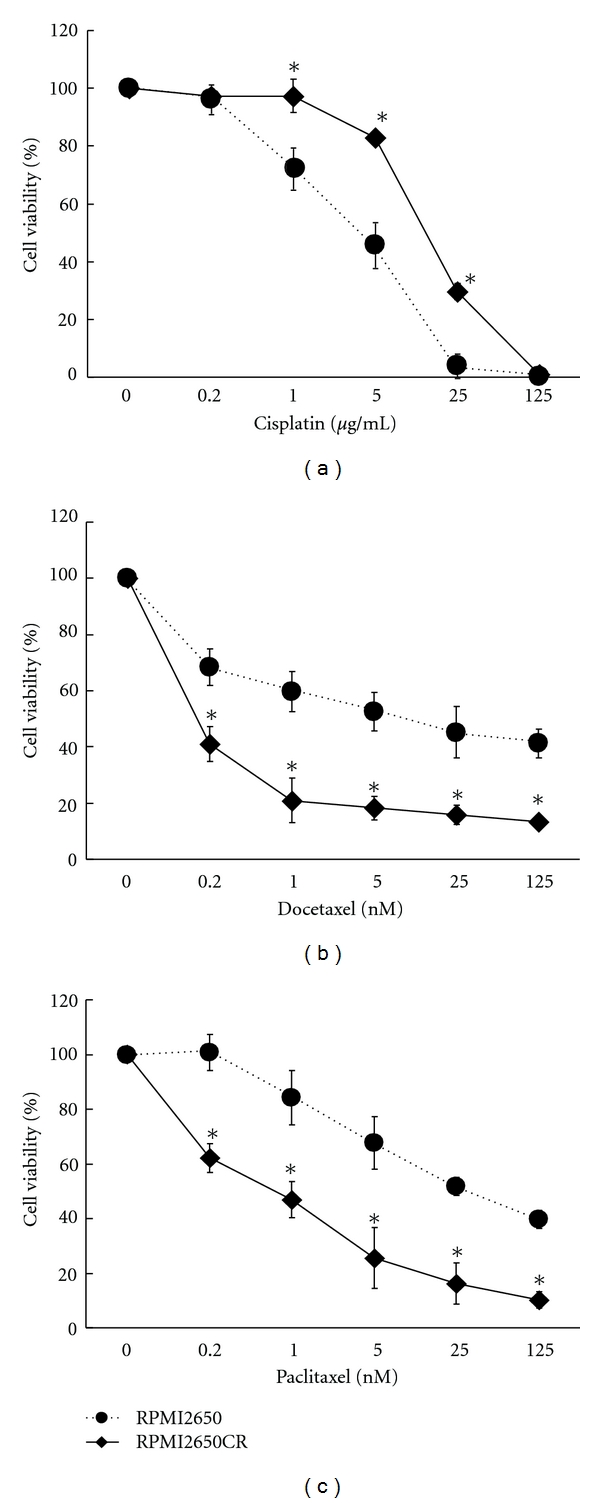
Cell viability with chemotherapeutic agents was assessed by MTT assay. RPMI2650CR shows higher resistance to cisplatin that the parental RPMI2650 (a) but has higher sensitivities to both docetaxel (b) and paclitaxel (c).

Stordal et al. reported an inverse correlation of chemosensitivity between taxane and CDDP [4]. Therefore, we further performed an MTT assay to elucidate taxane sensitivity using our established acquired CDDP-resistant HNSCC cell lines; 8 of the 9 analyzed CDDP-resistant cell lines showed sensitivities similar to their parental cell lines (data not shown), but the RPMI2650-derived CDDP-resistant cell line (RPMI2650CR) demonstrated hypersensitivity to taxanes when compared to the parental cell line (see Figure 1). It is notable that the IC50 value changed by one order of magnitude for CDDP chemosensitivity, but the IC50 values differed by two orders of magnitude in chemosensitivities to both taxanes. These phenomena were reproducible for the two different taxanes. Stably *IGF2*-expressing RPMI2650 was also used to determine the taxane sensitivity, but it showed a sensitivity similar to that of parental RPMI to both taxanes (data not shown), indicating that *IGF2 *is not involved in the hypersensitivity to taxanes. Although the molecular mechanisms behind the inverse correlation of chemosensitivity between taxane and CDDP are not well understood, it can be proposed that taxanes are good candidates as a second-line chemotherapeutic choice for HNSCC patients with acquired chemoresistance to CDDP. 

**Figure 2:**
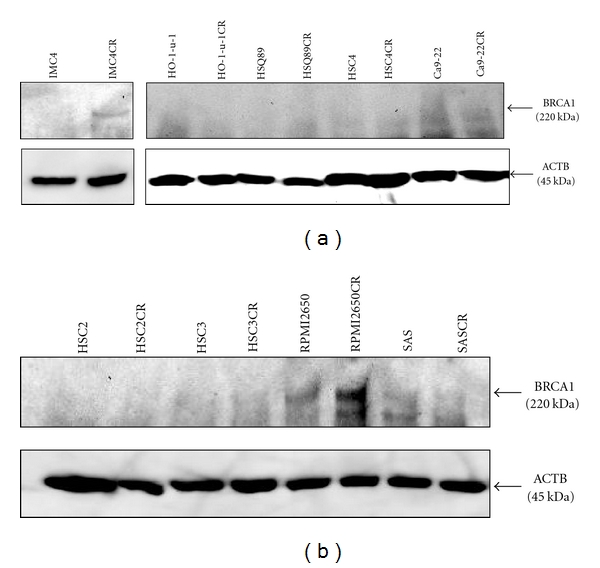
Western blot analysis of BRCA1 in HNSCC cell lines and their derived CDDP-resistant (CR) cell lines. Marked upregulation of BRCA1 (220 kDa) was observed only in the RPMI2650CR cell line. Beta actin (ACTB) was used as an internal control.

Several studies have suggested an association between taxane sensitivity and BRCA1 expression [8], so we then analyzed BRCA1 expression by Western blotting analysis in the 9 acquired CDDP-resistant cell lines we established. Results are shown in Figure 2; prominent upregulation of BRCA1 was observed in RPMI2650CR, and none other cell lines demonstrated a similar pattern. The possible clinical significance of BRCA1 detection is that preoperative immunostaining of a biopsy specimen for BRCA1 may indicate the effectiveness of chemotherapy with taxanes and may possibly additionally estimate CDDP sensitivity, ultimately leading to avoidance of the unnecessary employment of rich CDDP treatment, which has adverse side effects. Further studies are necessary to confirm the association of BRCA1 expression with chemotherapy resistance in HNSCC.

## 4. Conclusions

Acquired CDDP resistance is often observed in patients with HNSCC, and taxanes are used as the second-line chemotherapeutic drug for HNSCC. We observed that an acquired CDDP-resistant HNSCC cell line showed higher sensitivity to taxane by two orders of magnitude than its parental cell line. Prominent upregulation of BRCA1 was also observed in this CDDP-resistant/taxane-sensitive cell line. Expression status of BRCA1 can be a biomarker to forejudge the taxane sensitivity to patients with acquired CDDP-resistant HNSCC.
